# Reproductive history and blood cell telomere length

**DOI:** 10.18632/aging.101558

**Published:** 2018-09-19

**Authors:** Jacob K. Kresovich, Christine G. Parks, Dale P. Sandler, Jack A. Taylor

**Affiliations:** 1 Epidemiology Branch, National Institute of Environmental Health Sciences, NIH, Research Triangle Park, NC 27709, USA; 2 Epigenetic and Stem Cell Biology Laboratory, National Institute of Environmental Health Sciences, NIH, Research Triangle Park, NC 27709, USA

**Keywords:** telomeres, reproductive history, estrogen, qPCR

## Abstract

Telomeres are repetitive nucleotide sequences that protect against chromosomal shortening. They are replenished by telomerase, an enzyme that may be activated by estrogen. Women have longer telomeres than men; this difference might be due to estrogen exposure. We hypothesized that reproductive histories reflecting greater estrogen exposure will be associated with longer blood cell telomeres. Among women in the Sister Study (n= 1,048), we examined telomere length in relation to self-reported data on reproductive history. The difference between age at menarche and last menstrual period was used to approximate the reproductive period. Relative telomere length (rTL) was measured using qPCR. After adjustment, rTL decreased with longer reproductive period (β= -0.019, 95% CI: -0.04, -0.00, p= 0.03). Premenopausal women had shorter rTL than postmenopausal women (β= -0.051, 95% CI: -0.12, 0.01, p= 0.13). Longer breastfeeding duration was associated with longer rTL (β= 0.027, 95% CI: 0.01, 0.05, p=0.01); increasing parity was associated with shorter rTL (β = -0.016, 95% CI: -0.03, 0.00, p=0.07). Duration of exogenous hormone use was not associated with rTL. Reproductive histories reflecting greater endogenous estrogen exposure were associated with shorter rTL. Our findings suggest that longer telomeres in women are unlikely to be explained by greater estrogen exposure.

## Introduction

Telomeres are noncoding, hexanucleotide repetitive sequences that are located at the end of chromosomes. They serve as a buffer against end-to-end fusions, DNA damage checkpoint activation, and chromosomal shortening [1]. With each cell division, telomere length decreases due to incomplete end replication. Telomere shortening may be further influenced by endogenous factors including oxidative stress [2,3] and lifestyle factors such as multivitamin use, work schedule and physical activity [4–6]. Telomere length may additionally be used as a marker of disease risk [7–11] and may serve as a biomarker of the aging process [12].

Telomerase is the enzyme responsible for replenishing telomere length. It is generally active in cell-types with high replication rates, including germ, stem and immune cells [13,14]. Telomerase may be activated by estrogen; gene sequence analysis of the promoter region of telomerase reverse transcriptase (*TERT*) identified two estrogen response elements [15–17]. Estrogen-receptor alpha concentrations in hormone-sensitive tissues have been shown to mediate the relationship between estrogen and expression of *TERT* [18–20]. Moreover, estrogen deficiency in aromatase knockout female mice has been associated with decreased *TERT* expression resulting in shorter telomeres [21].

In human studies, women generally have longer telomeres than men [22,23]. Some studies suggest this sex difference may widen across the life course implying that women and men may have different rates of telomere erosion [24–27]. Life course estrogen exposure may explain these observations but there is little consensus across studies. Findings regarding long-term exogenous hormone use and blood cell telomere length are mixed [28–31]. Current endogenous estrogen production, estimated using menopausal status and circulating hormone concentrations, generally suggest inverse associations [32,33]. Notably, studies focused on endogenous estrogen generally have not accounted for potential exogenous sources.

Here, we examine the relationship between estrogen and telomere length using a life course approach that estimates exposure history to both endogenous and exogenous estrogen. Estrogen exposure in women can be approximated using characteristics of their reproductive histories. For example, reproductive period, calculated as the difference between age at menarche and last menstrual period, as well as other reproductive factors, may reflect endogenous estrogen production [34–36]. Birth control and hormone use duration can be used to estimate exogenous estrogen exposures. We hypothesize that among women, reproductive histories reflecting greater estrogen exposure over the life course will be associated with longer blood cell telomeres.

## RESULTS

Age was inversely associated with relative telomere length (rTL) quartile (*P*-trend< 0.01) (Table 1). After adjustment for participant age, older paternal age at birth was associated with increasing telomere length quartiles (*P*-trend= 0.05). Longer age-adjusted breastfeeding duration was associated with increasing relative telomere length quartiles (*P*-trend= 0.03).

**Table 1:** Age-standardized characteristics of Sister Study participants by quartile of telomere length (n= 1,048).

		Relative telomere length quartile				
		1 (shorter)	2	3	4 (longer)	*P*
						
Age (yr.)		58.1 ± 8.9	54.6 ± 8.8	54.0 ± 8.7	52.8 ± 9.2	< 0.01
White (%)		94.1	90.8	87.1	96.4	0.45
BMI (kg/m^2^)		27.8 ± 6.5	26.8 ± 5.2	27.6 ± 5.9	27.2 ± 5.6	0.90
Maternal age (yr.)		28.7 ± 5.9	28.5 ± 5.7	28.5 ± 6.1	29.3 ± 5.9	0.30
Paternal age (yr.)		32.0 ± 6.8	31.4 ± 6.6	32.2 ± 7.0	32.8 ± 7.0	0.05
Physical activity (METs/wk.)		48.2 ± 30.3	50.0 ± 30.9	54.4 ± 32.2	51.5 ± 29.5	0.15
Alcohol intake (drinks/wk.)		3.1 ± 4.8	2.9 ± 4.3	3.0 ± 4.5	2.9 ± 4.4	0.90
Smoking status						
Never (%)		53.3	54.0	55.3	56.6	0.51
Former (%)		38.1	38.1	38.2	35.6	0.61
Current (%)		8.6	7.9	6.5	7.8	0.75
Pack years		7.0 ± 12.7	6.3 ± 11.2	6.8 ± 12.5	7.2 ± 16.6	0.78
						
Reproductive period (yr.)		34.2 ± 6.9	33.2 ± 7.8	33.2 ± 7.3	33.3 ± 7.2	0.15
Premenopausal (%)		40.9	35.7	37.2	37.7	0.43
Hormone use (ever, %)^1^		68.4	68.5	67.7	67.4	0.78
Birth control pill (ever, %)		78.6	88.0	86.7	81.8	0.17
Menarche age (yr.)		12.5 ± 1.5	12.6 ± 1.4	12.6 ± 1.4	12.7 ± 1.4	0.26
Parity (total births)		2.0 ± 1.5	2.0 ± 1.3	1.9 ± 1.3	1.9 ± 1.4	0.21
Breastfeeding duration (yr.)		0.6 ± 1.1	0.6 ± 1.0	0.7 ± 1.1	0.9 ± 1.7	0.03
Breast cancer (%)		37.4	38.7	34.5	38.5	0.97


All values, except age, are age-standardized means ± standard deviations.

Abbreviations: BMI, body mass index; METs, metabolic equivalent tasks.

^1^ Restricted to postmenopausal women (n= 654)

In individual linear regression models, after adjusting for age, race, and paternal age, we found an inverse association with reproductive period (for each 5-year increase in duration of reproductive period, β_per 5-year_= -0.015, 95% confidence interval (CI)= -0.03, 0.00, *P*= 0.05) and a positive association with breastfeeding duration (β_per year_= 0.017, 95% CI: -0.00, 0.03, *P*= 0.06) (Table 2). Premenopausal women generally had shorter telomeres than postmenopausal women (β= -0.052, 95% CI: -0.12, 0.01, *P*= 0.11). After restriction to postmenopausal women, we similarly observed an inverse, though not statistically significant, association with age at menopause, such that women who transitioned to postmenopausal status later had shorter telomeres (data not shown).

**Table 2:** Adjusted associations between reproductive histories and relative telomere length, separate models (n= 1,048).

	β (95% CI)	p-value
Reproductive period (per 5 yrs.)	-0.015 (-0.03, 0.00)	0.05
Parity (per birth)	-0.007 (-0.02, 0.01)	0.40
Menopause status (pre- vs post)	-0.052 (-0.12, 0.01)	0.11
Breastfeeding (per yr.)	0.017 (-0.00, 0.03)	0.06
BC pill use (per 10 yrs.)	0.009 (-0.02, 0.04)	0.62
Hormone use (per 10 yrs.)^1^	0.003 (-0.03, 0.04)	0.86


Models adjusted for age at blood draw (yrs.), race/ethnicity (White, Black, Hispanic, Other), and paternal age (yrs.)

Abbreviation: birth control, BC.

^1^Analysis restricted to postmenopausal women (n= 654)

**Figure 1:**
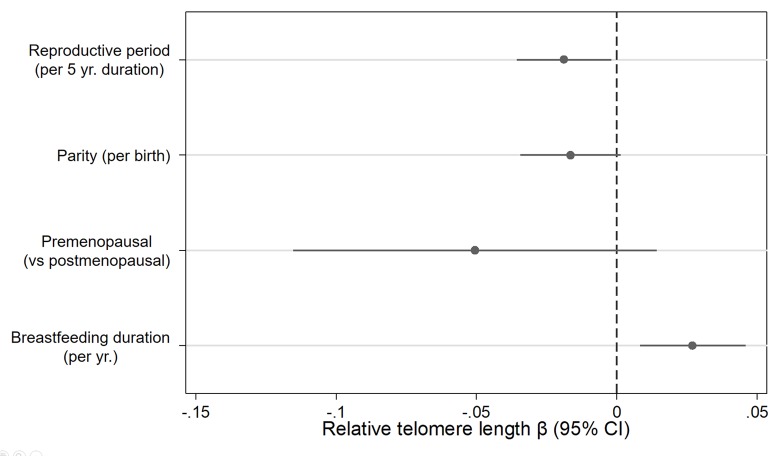
**Relationship between relative telomere length and reproductive histories associated with endogenous estrogen exposure.** Estimates derived from a model that included reproductive period (yrs.), parity (births), menopause status (pre- vs post), breast feeding duration (yrs.), age at blood draw (yrs.), race/ethnicity (White, Black, Hispanic, Other), paternal age (yrs.), and duration of hormone use (yrs.) and birth control pill use (yrs.).

**Figure 2:**
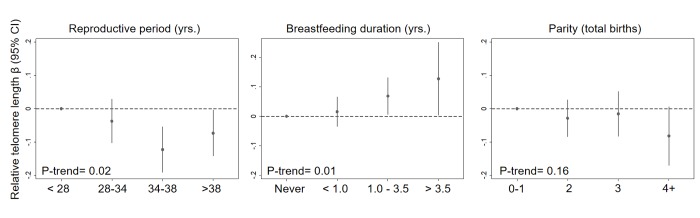
**Relationship between relative telomere length and reproductive histories associated with endogenous estrogen exposure in a mutually adjusted model by categories.** The model additionally adjusted for age at blood draw (yrs.), race/ethnicity (White, Black, Hispanic, Other), paternal age (yrs.), menopausal status (pre- vs post), and duration of hormone use (yrs.) and birth control pill use (yrs.).

In a single model with mutual adjustment for reproductive factors, we found inverse associations between telomere length and reproductive period (β_per 5 years_ = -0.019, 95% CI: -0.04, -0.00, *P*= 0.03), parity (β_per birth_= -0.016, 95% CI: -0.03, 0.00, *P*= 0.07) and premenopausal status (β= -0.051, 95% CI: -0.12, 0.01, *P*= 0.13) (Figure 1). We also observed a positive association between telomere length and breastfeeding duration (β_per year_ **=** 0.027, 95% CI= 0.01, 0.05, *P*= 0.01). The association with parity was strongest for women with four or more births relative to women with 0 or 1 births (β **= -**0.08, 95% CI= -0.17, 0.01, *P*= 0.07) (Figure 2). Finally, women who breastfed the longest (> 3.5 years) had longer telomeres than women who never breastfed (β **=** 0.13, 95% CI= 0.00, 0.25, *P*= 0.04). When we excluded women who had developed breast cancer before September 2016, associations remained consistent (Supplementary Table 1). Restriction to White women also did not appreciably change our results (Supplementary Table 2).

Findings using a replication sample are reported in Supplementary Table 3. Associations of rTL with reproductive period and breastfeeding were not replicated. Although the direction of the association was similar for our findings for parity, point estimates for all associations were weaker compared to estimates from the discovery population.

## DISCUSSION

Based on the observed sex differences in blood cell telomere length over the life course and known estrogen binding sites on telomerase, we hypothesized that reproductive histories representing greater life course estrogen exposure would be associated with longer blood cell telomeres. We found instead that longer reproductive periods (representing longer lifetime estrogen exposure) and premenopausal status (i.e. current estrogen exposure) were associated with shorter telomeres. Longer breastfeeding duration, a marker of reduced estrogen exposure [37], was associated with longer telomeres. We found exogenous hormone use did not influence blood cell telomeres. Our observed associations were contrary to our hypothesis and suggest life course estrogen exposure does not explain longer telomere length in women.

Our findings are, however, consistent with observations from other large cohort populations suggesting greater exposure to current endogenous estrogen may be associated with shorter telomere lengths. In the Nurse’s Health Study, De Vivo et al. (2009) found that higher circulating concentrations of estradiol and estrone were associated with shorter telomeres [32]. Dalgård et al. (2015) examined the rate of leukocyte telomere attrition among pre-, peri-, and postmenopausal women and found that, after accounting for age effects, the rate of attrition was fastest among premenopausal women (~20 base pairs/year vs ~15 base pairs/year) [33]. We also found additional support that exogenous hormone use may not be associated with blood cell telomere length [33,38]. To our knowledge, we are the first to report an association between telomere length and maternal breastfeeding history. Taken together, our findings suggest endogenous, but not exogenous, sources of estrogen may increase blood cell telomere erosion.

This inverse relationship may be explained by estrogenic stimulation of hematopoietic stem cells (HSC) [39,40]. HSCs give rise to blood cell subtypes including leukocytes and erythrocytes [41]. Interestingly, both erythrocyte turnover and HSC division may be regulated by estrogen [42]. Longer exposures to estrogen may therefore result in replacement of reduced erythrocyte populations by increasing HSC replication rates. As HSC telomeres shorten with each round of replication, subsequently differentiated blood cells may have shortened telomeres. This potential mechanism is supported by the finding that leukocyte and HSC telomere lengths are highly correlated [43]. This process has not been explicitly examined but supports the finding that longer estrogen exposure may result in replicative shortening of telomeres.

Although qPCR methods are optimized for measuring telomeres in large study populations, there are considerable limitations worth noting. Most importantly, assay reliability across study populations is generally limited due to technical variation [44]. qPCR also provides a relative estimate of telomere length which may be dependent on a reference population. Reference populations have not been standardized, making comparisons across studies difficult. qPCR additionally only provides an average telomere length measurement across all chromosomes resulting in non-specific measurements which may limit the ability to detect associations [45]. Finally, blood-based telomere length measurements are often derived from mixed cell populations which may introduce measurement error [46]. In our analysis, the replication population was oversampled for women who had high perceived stress, were current smokers, and non-White race. Additional model adjustment for these factors generally did not influence effect estimates or improve replication.

Sex differences in telomere length have been observed at birth suggesting telomere length may be established *in utero* [47,48]. It is therefore possible that reproductive history may only have modest effects on telomere length. Paternal age is a well-established, strong predictor of offspring telomere length at birth [48–51]. We adjusted for paternal age in our statistical models, thereby accounting for some inter-individual variation in telomere length. This allowed us to assess life course reproductive factor and telomere length associations more accurately. As reproductive factors are often related to each other, we used a single, mutually adjusted model to account for relationships between the reproductive factors which potentially results in more valid association estimates.

In summary, we observed inverse associations between blood cell telomere length and reproductive history characteristics representing both current and longer life course endogenous estrogen exposure. Although this finding suggests estrogen is not the driver of known sex differences in telomere length, it is consistent with prior reports and may suggest estrogenic stimulation of HSCs may outweigh upregulation of telomerase.

## METHODS

### Study population

The Sister Study is a prospective cohort study of 50,884 women designed to investigate environmental and lifestyle risk factors for breast cancer [52]. To be eligible for enrollment women had to be between 35 and 74 years old, have no history of breast cancer themselves, but have a sister who had been diagnosed with breast cancer. The breast cancer probands were not enrolled in the Sister Study. A case-cohort subsample of the study participants (n= 1,077) was previously selected to examine associations between whole blood cell telomere length and breast cancer risk and is described in detail elsewhere [53]. All women were cancer-free at the time of blood collection. Of the women who subsequently developed breast cancer, their blood samples were collected more than a year prior (mean= 460 days) to diagnosis and telomere length was not related to breast cancer risk [53]. Thus, the entire sample was included in this analysis. Participants gave written informed consent and the study protocol was approved by the NIEHS and Copernicus Group Institutional Review Boards.

### Reproductive history assessment

Information on reproductive life events was collected at study enrollment via a computer assisted telephone interview, including age at onset of menarche and last menstrual period. Information was also collected on reproductive behaviors associated with endogenous estrogen production including breastfeeding duration and parity, and exogenous estrogen exposure including duration of birth control pill and hormone use.

### Replication sample

We used an earlier, independent sample of Sister Study participants for replication of our findings. This study sample, drawn from early cohort participants (n= 647), was selected to examine associations between measures of stress and telomere length and was oversampled for women with high or very high perceived stress, current smokers, and non-Whites [54]. Forty-one women were tested in both the study population and the replication sample. To keep the replication sample independent from the main study sample, these women were excluded from the replication sample. Women missing exposure and covariate information were also excluded resulting in a final replication sample size of 597 participants.

### Telomere length measurements

Genomic DNA was extracted from whole blood samples collected at enrollment. rTL was measured using established qPCR methods as previously described [53]. Briefly, telomere length was measured as the relative ratio (T/S ratio) of telomere repeat copy number (T) to single gene copy number (S) using a monochrome, multiplex qPCR protocol. This method amplifies telomere and single genes in a single reaction tube; T/S ratios derived from this method correlate with terminal restriction fragment lengths measured by Southern blot [55]. A five-point standard curve was included in quadruplicate on each assay plate. Standard curve efficiencies for both T and S primers were above 90% and regression coefficients were at least 0.99 in all PCR runs. The average coefficient of variation was 11% and intra-class correlation coefficient of a single T/S ratio was 0.85. In the replication sample, the T/S ratio was assessed using an earlier qPCR protocol which employed separate reaction tubes to amplify telomere and single genes [56].

### Statistical analysis

Among a sample of women who were cancer free at blood draw, we conducted a cross-sectional study of estimated history of estrogen exposure and telomere length. We explored normality of estimated relative telomere length across the sample population using histograms and kernel density plots. Reproductive period was calculated as the difference between age at menarche and last menstrual period. We used linear and binary regression models to examine age-adjusted distributions of participant characteristics by relative telomere length quartiles. Trend across telomere quartiles was tested by treating telomere length quartile as an ordinal variable.

To examine associations between reproductive history characteristics and rTL we estimated beta values and 95% CIs using linear regression models adjusted for covariates. Telomere length was treated as a continuous variable. Reproductive period, parity, breastfeeding duration, and duration of birth control and hormone use were modelled continuously; menopausal status was treated as dichotomous. Although all the women in the study were cancer-free at time of enrollment, we also conducted a sensitivity analysis restricting the analysis to those who did not develop breast cancer through September 2016 (n= 664), the end of follow-up for the case-cohort analysis.

In all models, potential adjustment covariates included age at blood draw (yrs.), body mass index (kg/m^2^), race/ethnicity (White, Black, Hispanic, Other), smoking status (current, former, never), alcohol intake (drinks/wk.), maternal and paternal age at birth (yrs.), physical activity (metabolic equivalent tasks (METs)/wk.), and case-cohort subgroup (case/random cohort). Covariates were included in the model if they were significant predictors of blood cell telomere length. We included age at blood draw and race/ethnicity as *a priori* covariates. Paternal age at birth was selected as it was associated with telomere length in the study sample and is known predictor of relative telomere length [51]. In analyses exploring relationships with duration of hormone use, we restricted to postmenopausal women (n= 654). We examined associations between reproductive factors (reproductive period, parity, menopause status, breastfeeding duration, and birth control and hormone use) and telomere length both in individual models and in a single, mutually adjusted model. To reproduce findings from the discovery sample, we repeated all analyses in the replication subsample of participants. As the replication population was oversampled for women with high or very high perceived stress, current smokers, and non-Whites, we conducted additional analyses adjusting for the oversampled characteristics. We conducted all analyses among women with complete covariate information (n= 1,048) using Stata version 14.2 (College Station, TX).

### Data availability

Data access is restricted but can be made available upon reasonable request.

## Supplementary Material

Supplementary Tables
