# Supplementary material for: Reproductive history and blood cell telomere length
Source: Aging (Albany NY). 2018 Sep 19;10(9):2383–93. doi: 10.18632/aging.101558 (PMC6188490; doi:10.18632/aging.101558)
Supplement: Supplementary Tables [file aging-10-101558-s001.pdf]

SUPPLEMENTARY TABLES

Supplementary Table 1. Associations between reproductive history and relative telomere length among women who remained cancer free through September 2016 (n= 664).

	Individual models		Mutually adjusted model	
	β (95% CI)	p-value	β (95% CI)	p-value
Reproductive period (per 5 yrs.)	-0.014 (-0.03, 0.00)	0.13	-0.022 (-0.04, -0.00)	0.04
Parity (per birth)	-0.008 (-0.03, 0.01)	0.46	-0.018 (-0.04, 0.00)	0.11
Menopause status (pre- vs post)	-0.054 (-0.13, 0.02)	0.18	-0.058 (-0.14, 0.02)	0.15
Breastfeeding (per yr.)	0.016 (-0.00, 0.04)	0.12	0.026 (0.00, 0.05)	0.02
BC pill use (per 10 yrs.)	-0.003 (-0.04, 0.04)	0.87	0.004 (-0.04, 0.05)	0.86
Hormone use (per 10 yrs.)[1]	-0.018 (-0.06, 0.02)	0.40	-0.026 (-0.07, 0.02)	0.29

Models adjusted for age at blood draw (yrs.) and race (White, Black, Hispanic, Other), and paternal age (yrs.)
Abbreviation: birth control, BC
[1]Model restricted to postmenopausal women (n= 412)

Supplementary Table 2. Examining the impact of restriction to White women for the associations between reproductive histories and relative telomere length, separate models (n= 1,048).

	Original adjusted models n= 1,048			Restricted to White women n= 965	
	Individual models				
	β (95% CI)	p-value		β (95% CI)	p-value
Reproductive period (per 5 yrs.)	-0.015 (-0.03, 0.00)	0.051		-0.012 (-0.03, 0.00)	0.131
Parity (per birth)	-0.007 (-0.02, 0.01)	0.404		-0.005 (-0.02, 0.01)	0.583
Menopause status (pre- vs post)	-0.052 (-0.12, 0.01)	0.113		-0.042 (-0.11, 0.03)	0.230
Breastfeeding (per yr.)	0.017 (-0.00, 0.03)	0.056		0.019 (0.00, 0.04)	0.042
BC pill use (per 10 yrs.)	0.009 (-0.02, 0.04)	0.621		0.010 (-0.03, 0.05)	0.570
Hormone use (per 10 yrs.)[1]	0.003 (-0.03, 0.04)	0.860		-0.000 (-0.04, 0.04)	0.988
	Mutually adjusted models				
Reproductive period (per 5 yrs.)	-0.019 (-0.04, -0.00)	0.030		-0.017 (-0.04, 0.00)	0.059
Parity (per birth)	-0.016 (-0.03, 0.00)	0.071		-0.015 (-0.03, 0.00)	0.114
Menopause status (pre- vs post)	-0.051 (-0.12, 0.01)	0.126		-0.044 (-0.11, 0.02)	0.210
Breastfeeding (per yr.)	0.027 (0.01, 0.05)	0.005		0.029 (0.01, 0.05)	0.005
BC pill use (per 10 yrs.)	0.015 (-0.02, 0.05)	0.381		0.018 (-0.02, 0.05)	0.341
Hormone use (per 10 yrs.)[1]	-0.008 (-0.05, 0.03)	0.688		-0.011 (-0.05, 0.03)	0.590

"Original adjusted models" include age at blood draw (yrs.), race/ethnicity (White, Black, Hispanic, Other), and paternal age (yrs.). "Restriction to White women" models include age at blood draw (yrs.) and paternal age (yrs.)
Abbreviation: birth control, BC
[1]Model restricted to postmenopausal women (n= 654 in the full analyses; n= 606 after restriction to White women)

Supplementary Table 3. Mutually adjusted models for associations between reproductive history and relative telomere length (n= 597).

	Model 1		Model 2	
	β (95% CI)	p-value	β (95% CI)	p-value
Reproductive period (per 5 yrs.)	0.009 (-0.01, 0.03)	0.29	0.006 (-0.01, 0.02)	0.50
Parity (per birth)	-0.002 (-0.02, 0.01)	0.77	-0.002 (-0.02, 0.01)	0.77
Breastfeeding (per yr.)	-0.010 (-0.03, 0.01)	0.32	-0.013 (-0.03, 0.01)	0.22
Menopause status (pre- vs post)	0.006 (-0.05, 0.06)	0.85	-0.004 (-0.06, 0.06)	0.90
BC pill use (per 10 yrs.)	-0.003 (-0.03, 0.03)	0.86	-0.001 (-0.03, 0.03)	0.93
Hormone use (per 10 yrs.)[1]	0.012 (-0.02, 0.05)	0.51	0.011 (-0.03, 0.05)	0.56

Model 1 adjusted for age at blood draw (yrs.), race (White, Black, Hispanic, Other), and paternal age (yrs.)
Model 2 additionally adjusted for the oversampled characteristics (perceived stress, continuous; smoking status, current/former/never)
Abbreviation: birth control, BC
[1]Model restricted to postmenopausal women (n= 358)
